# Genome‐wide association studies reveal shared genetic haplotypes of autoimmune rheumatic and endocrine diseases with psychiatric disorders

**DOI:** 10.1002/brb3.2955

**Published:** 2023-03-16

**Authors:** Konstantinos Voskarides, Nefeli Giannopoulou, Rasha Eid, Konstantinos Parperis, Andreas Chatzittofis

**Affiliations:** ^1^ Department of Basic and Clinical Sciences University of Nicosia Medical School Nicosia Cyprus; ^2^ School of Veterinary Medicine University of Nicosia Nicosia Cyprus; ^3^ Medical School University of Cyprus Nicosia Cyprus; ^4^ Department of Clinical Sciences/Psychiatry Umeå University Umeå Sweden

**Keywords:** depression, genetic linkage, HLA, linkage disequilibrium, psoriasis, rheumatoid arthritis, schizophrenia

## Abstract

**Background:**

Several studies have shown that autoimmune diseases are associated with psychiatric diseases like depression and psychosis. Genetic evidence supports this association. The aim of this study was to investigate if genetic variants predisposing to autoimmune diseases and psychiatric disorders are genetically linked, constructing the common haplotypes.

**Methods:**

All registered single nucleotide polymorphisms (SNPs) in the Genome‐wide association studies (“GWAS catalog”) having been associated with autoimmune rheumatic and endocrine diseases were investigated for being in linkage disequilibrium with any psychiatric disorders’ associated SNPs. Analysis was performed by the LDtrait and LDhap bioinformatics tools.

**Results:**

Multiple chromosomal regions have been detected containing rheumatic/endocrine diseases’ predisposing SNPs and psychiatric disorders’ predisposing SNPs. The genetic haplotypes have been constructed for some of these genetic regions. Six of the autoimmune rheumatic and endocrine diseases examined here share a common haplotype with psychiatric diseases at the HLA locus 6p21‐22.

**Conclusion:**

Our study shows that autoimmune diseases and psychiatric diseases are genetically linked. Genetic haplotypes have been constructed, showing in detail this genetic linkage.

## Graphical abstract

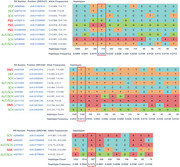

1

Several studies have shown that autoimmune diseases are associated with psychiatric diseases like depression and psychosis (Jeppesen & Benros, 2019). In addition, genetic evidence further supports this association (Tylee et al., 2022).

The aim of this study was twofold: (i) We investigated by linkage disequilibrium analysis all the genetic variants that have been associated with the most commonly studied autoimmune rheumatic and endocrine diseases, for any genetically linked variants/alleles predisposing to a psychiatric disease (genetic linkage is the tendency of nearby genetic alleles to be inherited together during the meiosis phase), (ii) By using the found linked genetic variants, we constructed genetic haplotypes (a genetic haplotype is a group of alleles inherited together from parents to children) that can potentially be useful for predicting the risk of a psychiatric disorder for individuals suffering from an autoimmune disease.

We downloaded all the single nucleotide polymorphisms (SNPs) that are registered in the “GWAS catalog” by the 23rd of February of 2022, with a *p*‐value 1 × 10^−8^ or lower, under the traits: Graves’ disease, Hashimoto's disease, diabetes mellitus type 1, psoriasis, ankylosing spondylitis, rheumatoid arthritis, systemic sclerosis (scleroderma), and Sjogren's syndrome, for genetic linkage investigation with any psychiatric disease (Table S1). We did not exclude any psychiatric traits for this investigation, but most associations in the GWAS catalog are associated with depression, anxiety, bipolar disorder, and schizophrenia. The PubMed ID and the first author of the GWAS studies that were used for our investigation are found in Table S1. We focused on autoimmune diseases with at least 30 registered associations in the GWAS catalog. Pediatric associations were excluded. Systemic lupus erythematosus and psychiatric comorbidities were investigated in a previous study conducted by our team (Parperis et al., 2022). The LDtrait tool (Lin et al., 2020) was used to detect the SNPs in linkage disequilibrium (*R* ^2^ ≥ 0.6). A 1 Mb window was set, upstream and downstream, for linkage disequilibrium analysis. The LDhap tool was used to identify and construct haplotypes containing risk alleles for autoimmune and psychiatric diseases (Machiela & Chanock, 2015). We did not exclude any populational ancestries from our study. On the other hand, most published GWAS studies have been performed in populations of European ancestry. In Table S1, haplotype frequencies can be found for all the human populations together and separately for the European ancestry populations.

The results are summarized in Table 1. To the best of our knowledge, Hashimoto's disease, Graves’ disease, ankylosing spondylitis, Sjogren's syndrome, and systemic sclerosis are investigated for the first time for common genetic haplotypes with any psychiatric disease. Evidence for genetic association between thyroid diseases and psychiatric diseases has been previously reported (Mo et al., 2019; Panicker, 2011). A genetic linkage of diabetes mellitus type 1 with schizophrenia and depression (Table 1) was revealed, an observation that has not been shown by previous studies (Tylee et al., 2022). This association is compatible with published epidemiological studies (Chen et al., 2022). We also found a genetic linkage between Graves’ disease, psoriasis, rheumatoid arthritis, and Sjogren's disease with psychosis and mood disorders, justified by previous epidemiological studies (Jeppesen & Benros, 2019). Three SNPs were found to have a pleiotropic effect (Table 1), predisposing for both an autoimmune and a psychiatric disease (rs4622308 predisposes for rheumatoid arthritis and anorexia nervosa, and two nearby SNPs for schizophrenia and diabetes mellitus type 1, respectively). Based on these findings, we constructed genetic haplotypes, including risk alleles for autoimmune and psychiatric diseases. Unfortunately, the risk alleles are not known for all the registered SNPs in the GWAS catalog, a limitation for constructing all the haplotypes of the chromosomal regions (Table 1). We finally constructed 10 risk haplotypes (Table 1 and Table S1). Some of the constructed haplotypes have a very high frequency in the general population. Notably, these genetic haplotypes cannot explain the high comorbidity of the autoimmune diseases with psychiatric phenotypes alone, but they are just a fraction of this complex phenotype.

**TABLE 1:** Genetic loci containing linked SNPs, predisposing for autoimmune and psychiatric diseases

Autoimmune disease	Linked psychiatric disease (chromosomic region) (flanking SNPs)	Constructed genetic haplotypes (frequency)	Pleiotropic SNPs (diseases)
Hashimoto's disease	None found	–	–
Grave's disease	Anxiety (16p11.2) (rs8050588‐rs3751855) Depression (20q13.12) (rs12624433‐rs1569723) Autism spectrum disorder, schizophrenia (6p21.31‐32) (rs6457617‐rs4713693)	16p11.2 (43%) 20q13.12 (1.8%) 6p21.31‐32 (29%)	–
Diabetes mellitus type 1	Schizophrenia, anorexia nervosa (12q13.2) (rs1689510‐rs4622308) Depression (15q14) (rs72727394‐rs56059718) Bipolar disorder (16p11.2) (rs151181‐rs4788084) Autism spectrum disorder, schizophrenia (17q21.31) (rs1052553‐rs199503) Schizophrenia, ADHD (19q13.33) (rs516246‐rs603985) Depression, autism spectrum disorder, schizophrenia (6p21.32‐p22.2) (rs4320356‐rs2647044)	12q13.2 (0.28%) 6p21.32‐p22.2 (22%)	–
Ankylosing spondylitis	Schizophrenia, ADHD (19q13.33) (rs679574‐rs603985) Depression (3p21.31) (rs4625‐rs3197999)	–	–
Psoriasis	Anxiety (16p11.2) (rs4889526‐rs59735493) Schizophrenia, ADHD (19q13.33) (rs679574‐rs603985) Depression (3p21.31) (rs4625‐rs3197999) Depression, autism spectrum disorder, schizophrenia (6p21.33) (rs6906565‐rs3130614)	16p11.2 (40%) 6p21.33 (3.55%)	rs10761661 (psoriasis and diabetes mellitus type 2, bipolar disorder) Nearby genes: *ALDH7A1P4, ADO*
Rheumatoid arthritis	Schizophrenia (2q11.2) (rs6712515‐rs9653442) Depression (20q13.12) (rs12624433‐rs4810485) Anxiety (5q21.1) (rs2561477‐rs391236) Autism spectrum disorder, schizophrenia (6p21.32‐33) (rs2596500‐rs9275511)	6p21.32‐33 (14%)	rs4622308 (rheumatoid arthritis, anorexia nervosa) Nearby genes: *ERBB3, RPS26* rs6712515 (rheumatoid arthritis, schizophrenia) Nearby genes: *AFF3*
Systemic sclerosis (scleroderma)	Autism spectrum disorder, schizophrenia (6p21.32) (rs3129763‐rs9275511)	6p21.32 (34%)	–
Sjogren's syndrome	Depression, autism spectrum disorder, schizophrenia (6p21.32‐33) (rs3131010‐rs2647044)	6p21.32‐33 (0.18%)	–

Abbreviation: snps, single nucleotide polymorphisms.

All genes lying in the constructed haplotypes can be found in Table S1. Six of the autoimmune rheumatic and endocrine diseases examined here share a common haplotype with psychiatric diseases at the HLA locus 6p21‐22 (Figure 1 and Table 1). This chromosomal region is associated with immune response and autoimmunity. Interestingly, by comparing all the autoimmune/psychiatric combined 6p21‐22 haplotypes, their common region is close to the *HLA‐DQB1* gene. Genetic variants inside or close to the *HLA‐DQB1* gene have been previously associated with psychiatric and autoimmune diseases. Further investigation of this gene could potentially reveal more genetic clues between autoimmunity and psychiatric traits. For example, this gene could be sequenced in patients exhibiting autoimmune and psychiatric traits, examining for any comorbidity‐related risk variants.

**FIGURE 1:**
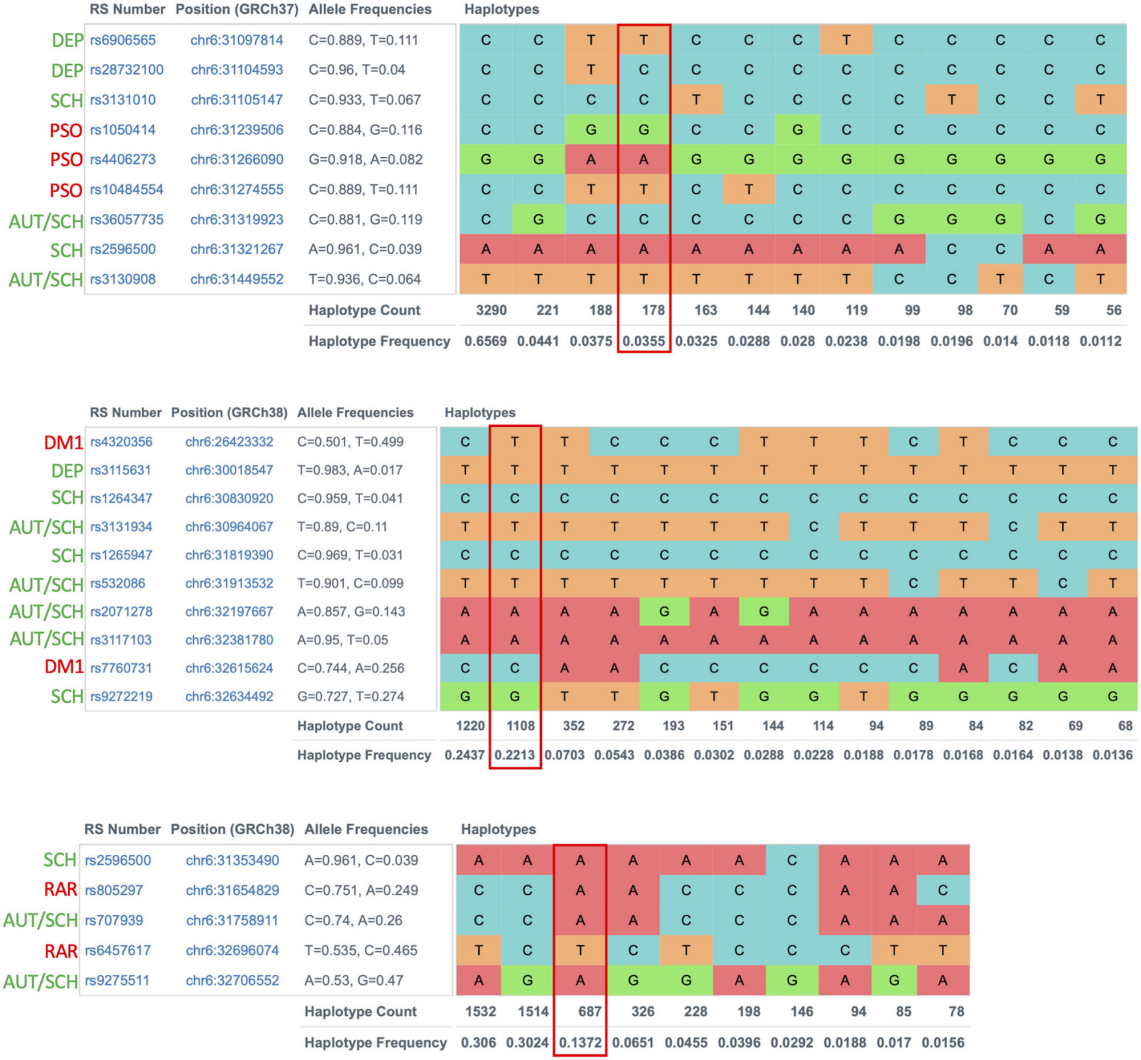
Genetic haplotypes on chromosomal region 6p21‐22 containing risk alleles for autoimmune and psychiatric diseases. Note: The risk haplotype is marked with a redrectangular. Abbreviations: AUT, autism; DEP, depression; DM1, diabetes mellitus type 1; PSO, psoriasis; RAR, rheumatoid arthritis; SCH, schizophrenia.

Our study shows that autoimmune diseases and psychiatric diseases are genetically linked. The construction of risk haplotypes has shown in detail this genetic linkage. This observation highlights the significance of comorbidity investigation under the view of genetic linkage. We plan to continue our research efforts by aiming to generate more genetic haplotypes that could be combined with environmental factors, in order to predict more accurately the risk of developing psychiatric comorbidities in patients with autoimmune diseases.

## FUNDING INFORMATION

Not any special funding was received for this research.

### PEER REVIEW

The peer review history for this article is available at https://publons.com/publon/10.1002/brb3.2955

## Supporting information

Table S1. Genetic variants, linkage disequilibrium data, and genetic haplotypes with all the included genes, for the diseases analyzed in this study.

## Data Availability

The data that support the findings of this study are inside the paper and in the accompanied Supplementary Material (online).

## References

[brb32955-bib-0001] Chen, M. H. , Tsai, S. J. , Bai, Y. M. , Huang, K. L. , Su, T. P. , Chen, T. J. , & Hsu, J.‐W. (2022). Type 1 diabetes mellitus and risks of major psychiatric disorders: A nationwide population‐based cohort study. Diabetes & Metabolism, 48, 1–6. 10.1016/j.diabet.2022.101319 35026379

[brb32955-bib-0002] Jeppesen, R. , & Benros, M. E. (2019). Autoimmune diseases and psychotic disorders. Frontiers in Psychiatry, 10, 1–11. 30949074 10.3389/fpsyt.2019.00131 PMC6435494

[brb32955-bib-0003] Lin, S. H. , Brown, D. W. , & Machiela, M. J. (2020). LDtrait: An online tool for identifying published phenotype associations in linkage disequilibrium. Cancer Research, 80, 3443–3446. 32606005 10.1158/0008-5472.CAN-20-0985 PMC7442674

[brb32955-bib-0004] Machiela, M. J. , & Chanock, S. J. (2015). LDlink: A web‐based application for exploring population‐specific haplotype structure and linking correlated alleles of possible functional variants. Bioinformatics, 31, 3555–3557. 26139635 10.1093/bioinformatics/btv402 PMC4626747

[brb32955-bib-0005] Mo, D. , Li, J. , Peng, L. , Liu, Z. , Wang, J. , & Yuan, J. (2019). Genetic polymorphisms on 4q21.1 contributed to the risk of Hashimoto's thyroiditis. Genetic Testing and Molecular Biomarkers, 23, 837–842. 31750736 10.1089/gtmb.2019.0125

[brb32955-bib-0006] Panicker, V. (2011). Genetics of thyroid function disease. Clinical Biochemist Reviews, 32, 165–175. 22147956 PMC3219766

[brb32955-bib-0007] Parperis, K. , Kyriakou, A. , Voskarides, K. , & Chatzittofis, A. (2022). Suicidal behavior in patients with systematic lupus erythematosus: Systematic literature review and genetic linkage disequilibrium analysis. Seminars in Arthritis and Rheumatism, 54, 1–9. 10.1016/j.semarthrit.2022.151997 35344734

[brb32955-bib-0008] Tylee, D. S. , Lee, Y. K. , Wendt, F. R. , Pathak, G. A. , Levey, D. F. , De Angelis, F. , Gelernter, J. , & Polimanti, R. (2022). An atlas of genetic correlations and genetically informed associations linking psychiatric and immune‐related phenotypes. JAMA Psychiatry, 79, 667–676. 10.1001/jamapsychiatry.2022.0914 35507366 PMC9069342

